# Uniform Sampling of Steady States in Metabolic Networks: Heterogeneous Scales and Rounding

**DOI:** 10.1371/journal.pone.0122670

**Published:** 2015-04-07

**Authors:** Daniele De Martino, Matteo Mori, Valerio Parisi

**Affiliations:** 1 Center for life nanoscience CLNS-IIT, P.le A.Moro 2, 00185, Rome, Italy; 2 Dipartimento di Fisica, Sapienza Universitá di Roma, P.le A.Moro 5, 00185, Rome, Italy; University of Glasgow, UNITED KINGDOM

## Abstract

The uniform sampling of convex polytopes is an interesting computational problem with many applications in inference from linear constraints, but the performances of sampling algorithms can be affected by ill-conditioning. This is the case of inferring the feasible steady states in models of metabolic networks, since they can show heterogeneous time scales. In this work we focus on rounding procedures based on building an ellipsoid that closely matches the sampling space, that can be used to define an efficient hit-and-run (HR) Markov Chain Monte Carlo. In this way the uniformity of the sampling of the convex space of interest is rigorously guaranteed, at odds with non markovian methods. We analyze and compare three rounding methods in order to sample the feasible steady states of metabolic networks of three models of growing size up to genomic scale. The first is based on principal component analysis (PCA), the second on linear programming (LP) and finally we employ the Lovazs ellipsoid method (LEM). Our results show that a rounding procedure dramatically improves the performances of the HR in these inference problems and suggest that a combination of LEM or LP with a subsequent PCA perform the best. We finally compare the distributions of the HR with that of two heuristics based on the Artificially Centered hit-and-run (ACHR), *gpSampler* and *optGpSampler*. They show a good agreement with the results of the HR for the small network, while on genome scale models present inconsistencies.

## Introduction

The metabolism of cells is based on a complex metabolic network of chemical reactions performed by enzymes, which are able to degrade nutrients in order to produce biomass and generate the energy needed to sustain all other tasks the cell has to perform [1]. The high-throughput data coming from genome sequencing of single organisms can be used to reconstruct the complete set of enzymes devoted to metabolic functions, leading to models of metabolism at the scale of the whole genome, whose analysis is computationally challenging [2]. If we want to model a metabolic system in terms of the dynamics of the concentration levels, even upon assuming well-mixing (no space) and neglecting noise (continuum limit), we have a very large non-linear dynamical system whose parameters are mostly unknown. For a chemical reaction network in which *M* metabolites participate in *N* reactions (where *N*,*M* ≃ 𝒪(10^2–3^) in genome-scale models) with the stoichiometry encoded in a matrix **S** = {*S*_*μr*_}, the concentrations *c*_*μ*_ change in time according to mass-balance equations

$$\begin{array}{c} \dot{c}=S\cdot v \end{array}$$ (1)

where *v*_*i*_ is the flux of the reaction *i* that in turn is a possibly unknown function of the concentration levels *v*_*i*_(**c**), with possibly unknown parameters. A simplifying hypothesis is to assume the system in a steady state **ċ** = 0. The fluxes are further bounded in certain ranges $v_{r}\in [v_{r}^{\text{min}},v_{r}^{\text{max}}]$ that take into account thermodynamical irreversibility, kinetic limits and physiological constraints. The set of constraints

$$\begin{array}{ccc} & & S\cdot v=0,\\ & & v_{r}\in \left[v_{r}^{\min},v_{r}^{\max}\right] \end{array}$$ (2)

defines a convex closed set in the space of reaction fluxes: a polytope from which feasible steady states should be inferred.

In general, the problem of the uniform sampling of convex bodies in high dimensions is both of theoretical and practical importance. From a theoretical viewpoint it leads to polynomial-time approximate algorithms for the calculation of the volume of a convex body [3], whose exact determination is a #P-hard problem [4]. On the other hand general problems of inference from linear constraints require an uniform sampling of the points inside a convex polytope: other examples apart from metabolic network analysis [5] include compressed sensing [6], freezing transition of hard spheres [7] and density reconstruction from gravitational lensing in astrophysics [8]. The knowledge of all the vertices characterizes completely a polytope but deterministic algorithms that perform an exhaustive enumeration can be infeasible in high dimensions since the number of such vertices could scale exponentially with the dimension. An alternative is to carry out a statistical analysis of the space by means of Monte Carlo methods [9]. A static approach with a simple rejection rule is feasible for low dimensions [10] but we have to recur to dynamical methods in high dimensions. The faster and most popular algorithm in order to sample points inside convex bodies is the hit–and–run (HR) Markov Chain Monte Carlo[11, 12].

**Fig 1:**
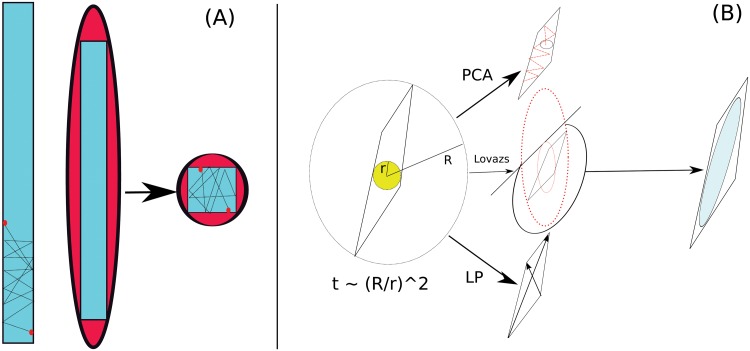
Sketch of the ill–conditioning problem in the uniform sampling from the polytope of metabolic steady states (A). We propose to build a matching ellipsoid in three ways (B): PCA, LP and LEM: see section Materials and methods for a full description.

The mixing time of the HR, that is the time to converge to the desired distribution, it scales as a polynomial of the dimensions of the body but the method can suffer of ill–conditioning if the body is highly heterogeneous as we sketch in Fig 1 (A). More precisely the mixing time *τ* scales like [13]

$$\begin{array}{c} \tau ≃𝒪(D^{2}R^{2}/r^{2}) \end{array}$$ (3)

where *D* is the dimension of the polytope, *R*,*r* are the radii of respectively the minimum inscribing and the maximum inscribed balls: *R*/*r* has been called the sandwiching ratio of the body. The sandwitching ratio quantifies the degree of ill–conditioning of the sampling problem and we will refer to it as its condition number.

Several alternatives have been proposed to the HR dynamics. A simple one consists in a rather coarse approximation: a certain number of vertices is calculated by linear programming applied to random linear objective functions and the points inside can be sampled by interpolation. This approximation suffers from the fact that we are neglecting possibly an exponentially large number of vertices, and it has been shown that this leads to wrong results even for simple hypercubes [8]. Artificially Centered hit–and–run (ACHR) [14], is a non-markovian modification of the HR algorithm that uses previously sampled points to determine the elongated directions of the space. ACHR has been widely used in order to sample flux configurations in metabolic networks [15–17] but it has the drawback that its non-markovian nature doesn’t guarantee the convergence to an uniform distribution. Finally, the sampling problem has been reformulated within the framework of Message Passing (MP) algorithms[18, 19], which allow very fast sampling, but work under the approximation of a tree-like network and are not guaranteed in general to converge to an uniform distribution. On the other hand it is known that the sandwitching ratio of a polytope can be reduced to at most $\sqrt{D}$ for centrally symmetric polytopes and to *D* in general, by an affine transformation defined by the so-called Loewner–John Ellipsoid [20], i.e. the ellipsoid of maximal volume contained in the polytope. Unfortunately this ellipsoid cannot be found in polynomial time, but it has been shown by L. Lovazs that a weaker form of the Loewner–John ellipsoid, with a factor of *D* ^3/2^, can be found in polynomial time [21] The feasible steady states of a metabolic network can show very heterogeneous scales on genome-scale models: previous samplings [22] seem to indicate that the distribution of flux scales can span 5 orders of magnitudes, and we should thus expect *R*/*r* ≃ 10^5^ in practical cases, that means that the ill–conditioning is a crucial issue in this inference problem. The focus of this work is on the reduction of the condition number in the uniform sampling of convex polytopes by finding an ellipsoid that closely matches the underlying space. We use this matching ellipsoid to extract the direction of the HR, a procedure that is equivalent to an affine transformation and eliminates the ill–conditioning. We remind that an affine transformation would keep the uniformity of the sampling since the Jacobian is constant.

We will analyze and compare three methods: the first is based on building an ellipsoid by applying principal component analysis (PCA) to previous samplings, the second, inspired by a technique called *Flux Variability Analysis* (FVA), uses linear programming (LP) in order to calculate the axes of the ellipsoid by maximizing and minimizing repeatedly the constraints defining the polytope, and finally the Lovazs ellipsoid method [21] (see Fig 1 (B) for a sketch).

We will focus on the problem of characterizing the space of feasible steady states in three networks of growing size: the catabolic core of the reconstruction of the metabolism of the bacterium Escherichia coli iAF1260 [23] and two genome scale models, respectively of *Saccaromyces Cerevisiae* (SCiND750) [24] and cervix squamous epithelial cells [25] (CERVIX). We then compare our uniform sampling with the results provided by two ACHR-based heuristics provided with the COBRA toolbox *gpSampler* [15] and *optGpSampler* [16]. The description of the methods, i.e. the construction of ellipsoids that matches the space by means of PCA, LP and Lovazs method follows thereafter.Finally we draw out some conclusions and perspectives.

## Results

**Fig 2:**
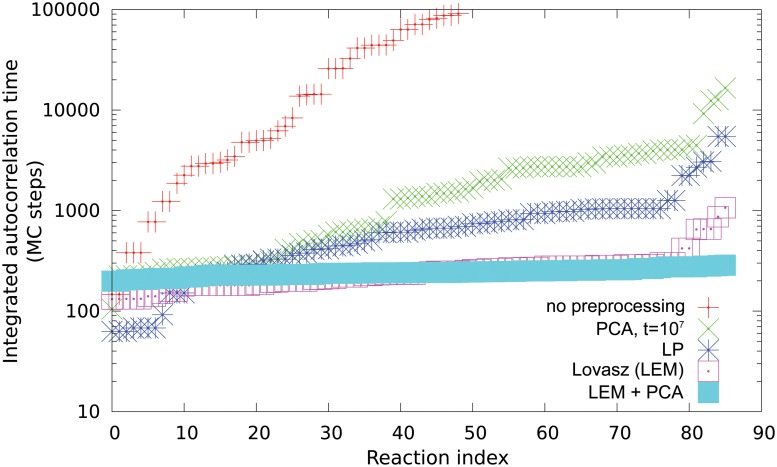
Integrated autocorrelation times ordered for increasing values of the steady state fluxes of the core model *E. coli* iAF1260 during an hit–and–run dynamics. Several preprocessing rounding procedures are compared: none, PCA, LP, LEM, LEM+PCA.

We first discuss the application of the rounding methods in order to sample the feasible steady states of the *E. coli*’s metabolic network reconstruction iAF1260 catabolic core. This a network with *M* = 72 metabolites and *N* = 95 reactions, including all exchange reactions. We consider the flux bounds provided with the model and employ bounds for the exchange fluxes that include possibility to intake the main elements that are needed in order to produce biomass: glucose, oxygen, ammonia, water and phosphate. Upon deleting null reactions we are left with a network of *M* = 68 metabolites and *N* = 86 reactions. The resulting polytope has *D* = 23 dimensions. In Fig 2 we report the integrated autocorrelation times of the fluxes during the HR with different pre-processing schedules, ordered for increasing values. The measure of integrated autocorrelation times is a rather standard procedure in order to asses the reliability of average estimates in Markov chains, we refer to the supplementary materials for further details. The autocorrelation time is an approximate measure of the number of steps after which the samples become independent. In Table 1 we report the machine time needed to obtain the rounding ellipsoid and the measured maximum integrated autocorrelation time among the sampled reaction fluxes. The times for the algorithm without preprocessing are very large, i.e. billions of Monte Carlo steps (hours for our implementation) in order to obtain reliable estimates for the flux averages. The preprocessing with PCA alone improves the situation, but the attainment to stationarity of the covariance matrix is still lacking. The LP and LEM methods alone successfully reduce the condition number rendering the sampling possible in feasible computational times. In particular the Lovazs method performs better in this case. Finally, the best result that minimizes the integrated autocorrelation times comes upon combining LEM method (or LP) with a subsequent PCA. In this way it is possible to obtain the ellipsoid directly from a good estimator of the stationary connected covariance matrix. Once the polytope has been rounded with a matching ellipsoid the mixing time of the HR Markov chain scales as a polynomial of the system size and it would be possible to perform a rigorous uniform sampling even of genome scale network models, whose number of reactions is typically of the order of thousands. We have thus performed our preprocessing and subsequent sampling of two genome scale models, i.e. the model for *Saccaromices Cerevisiae* SCiND750 [24] and a model of cervix squamous epithelial cells (CERVIX) from the reconstruction Recon 2 [25]. We consider the first with default bounds for the uptakes while for the second we leave the network completely open to test the different cases. After removal of blocked reactions, the resulting dimensions of the polytope are *D* = 180 and *D* = 694, respectively. It turns out that with our implementation the more convenient rounding procedure consists in using the LP approach with a subsequent PCA. The procedure is intensive but feasible, it requires approximately 30m to find an ellipsoid for SCiND750 and 3h for CERVIX, for a sake of comparison the Lovazs method requires 15h for SCiND750. From the analysis of integrated autocorrelation times we get a maximum value in MC steps of the order of 10^4^ where a MC step can performed in milliseconds, as it is summarized in Table 2.

**Table 1:** Preprocessing time and maximum integrated autocorrelation time for the hit–and–run algorithms being examined on the *E. coli* core iAF1260 metabolic network. On an Intel dual core at 3.06*GHz* using a single thread.

Time	Normal	PCA	LP	LEM	LEM(LP)+PCA
Preprocessing time (s)	0	40	7	4	4.2 (7.2)
Max.int.autocor.time (mc steps)	𝒪(10^9^)	1.6⋅10^4^	5.4⋅10^3^	1.1⋅10^3^	285

**Table 2:** Time performance of our implementation of the hit–and–run on genome scale networks. On an Intel dual core processor with clock rate 3.06*GHz* using a single thread.

Time	SciND750	CERVIX
Preprocessing time (h)	0.5	3
Max.int.autocor.time (mc steps)	1.6⋅10^4^	7.4⋅10^4^
Average time for one mc step (ms)	2	8

**Fig 3:**
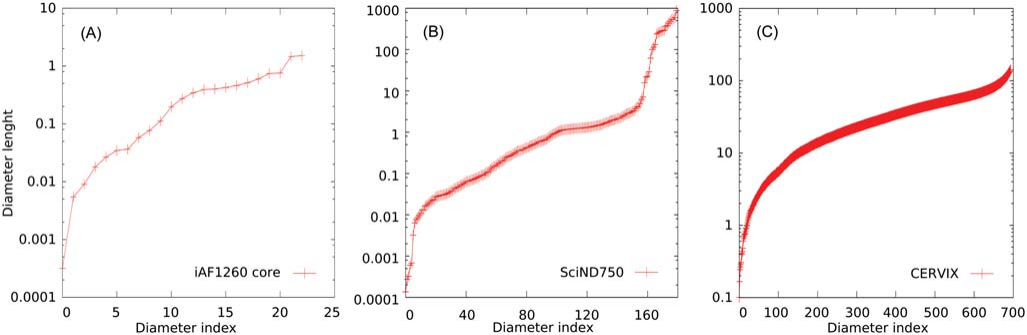
Diameters, ordered for increasing length, of the ellipsoids retrieved from the models iAF1260 Core (A), SciND750 (B) and CERVIX (C). Ellipsoids are obtained from the diagonalization of the stationary connected covariance matrix calculated by combining LP and PCA.

We can characterize heterogeneity of scales by looking at the length of the diameters obtained by diagonalizing the covariance matrix of the ellipsoid, obtained with a combination of LP (or Lovasz) and a subsequent PCA in order to guarantee the best approximation of the Loewnwer-John ellipsoids, which we show in Fig 3: even the small network (A) spans across four order of magnitude, while the genome scale network SCiND750 (B) spans across eight orders of magnitude. We remind that, at odds with the general case of a convex body, the calculation of the diameters of an ellipsoid consist exactly in this diagonalization task, that is a feasible problem of linear algebra. This strong heterogeneity would affect dramatically the performances of montecarlo markov chains without some pre-processing. On the other hand, the largest CERVIX network, being completely open, it spans across three order of magnitude in a continuous fashion.

### Comparison with artificially centered hit–and–run based methods

We have thus seen that a rigorous uniform sampling of steady states in genome-scale metabolic networks is feasible, albeit intensive, with the HR algorithm once the ill–conditioning has been removed with a rounding procedure. We can use here our results to test the validity of ACHR-based heuristics, that can be used with the COBRA [15] toolbox, *gpSampler* [15] and *optgpSampler* [16]. We have checked that the two ACHR methods give very similar distributions upon waiting an effective convergence of *gpSampler*, that for the largest network analyzed requires around 1 week of machine time on an Intel dual core at 3.06 GHz using a single thread. We acknowledge on the other hand that *optGpSampler* converges in much shorter times, and it is faster of the HR with our implementation once the rounding preprocessing time is taken into account, we report in Table 3 the machine times. On the small *E. coli* Core network half of the marginal flux distributions retrieved by ACHR based methods are consistent with the ones obtained by the HR according to the Kolmogorov-Smirnov test (KS) [26] with a confidence of 5%.

**Table 3:** Machine time, on an Intel dual core at 3.06*GHz* using a single thread, in order to retrieve 2⋅10^4^ points with rate *k* = 5000 by *optGpSampler* or with mixing fraction < 0.55 by *gpSampler*.

Method	iAF1260 Core	SciND750	CERVIX
*gpSampler*	20m	3d	8d
*optGpSampler*	2m	1h	5h

**Fig 4:**
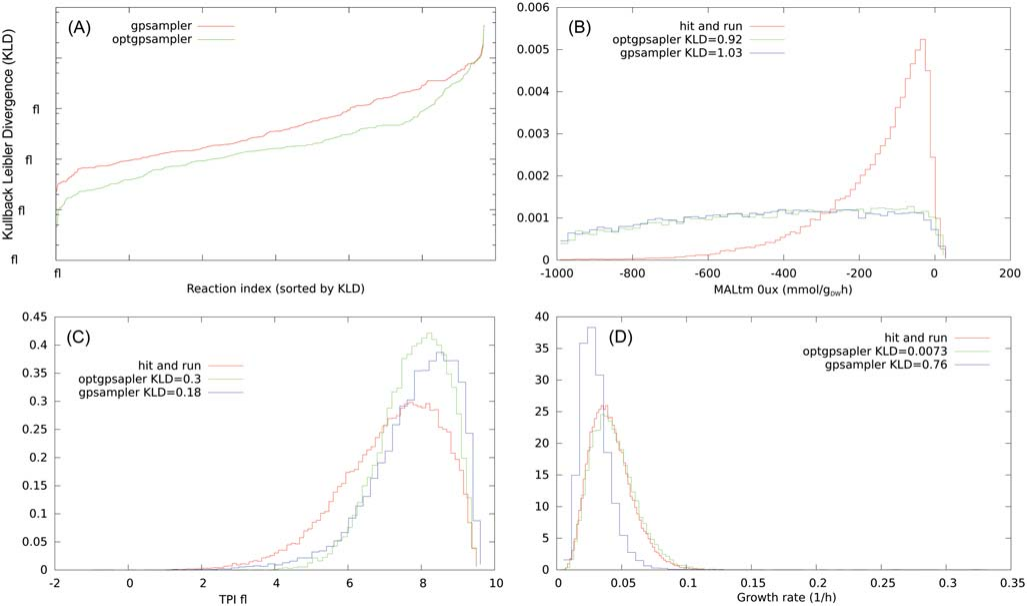
Consistency test of *gpSampler* and *optGpSampler* with the hit–and–run on the model SciND750. (A): KL divergence values of the marginal distributions of non null fluxes for *gpSampler* (red) and *optGpSampler* (green) compared with the hit–and–run, ordered for increasing values. (B): Marginal distributions of MALtm, this is a case in which *KLD* > 0.5 (5% of the cases for *optGpSampler*). (C): Marginal distributions of TPI, this is a case in which 0.05 ≤ *KLD* ≤ 0.5 (15% of the cases for *optGpSampler*). (D): Marginal distributions of the growth rate, this is a case in which for *optGpSampler* *KLD* < 0.05 (80% of the cases for *optGpSampler*). Histograms are obtained from 2⋅10^4^ points.

**Fig 5:**
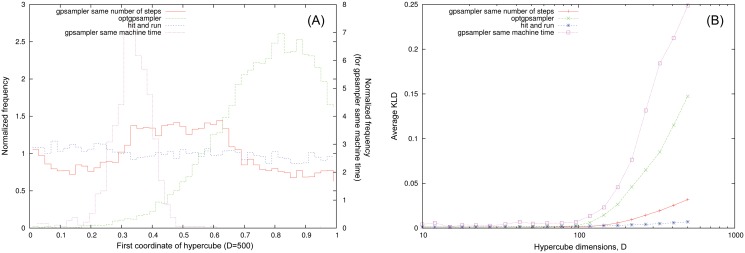
Consistency test of *optGpSampler* and *gpSampler* on hypercubes. (A): Marginal distributions of the first coordinate for *D* = 500 for the hit and run, gpsampler (same machine time and same number of steps) and optgpsampler. (B): Average value of the KLD over the coordinates with respect to the flat distribution as a function of the hypercube dimension.

The remaining marginal flux distributions are not in rigorous agreement but they provide a reliable approximation as it can be seen by the low values of the Kullback-Leibler divergence (KLD). If we have two distributions *P*(*x*) and *Q*(*x*) the KLD is defined as

$$\begin{array}{c} KLD\left(Q|P\right)=\int dxP\left(x\right)\log_{2}\left(P\left(x\right)/Q\left(x\right)\right), \end{array}$$ (4)

it is measured in *bits* and it quantifies the information that is lost by approximating *P* with *Q*. More precisely, if we extract *N* points from *Q* we would be *deceived* with probability 2^−*N*⋅*KLD*(*Q*∣*P*)^, i.e. we would believe the points come from *P* [27]. On the genome scale networks the marginal distributions retrieved by ACHR based methods do not pass the KS test, but they give an approximation. We have classified the level of approximation according to the value of the KLD with respect to the distribution retrieved by the HR algorithm. For instance in SciND750, the flux distributions retrieved by *optGpSampler* have *KLD* < 0.05 in 80% of the cases (good agreement), 0.05 ≤ *KLD* ≤ 0.5 in 15% of them (approximate) and *KLD* > 0.5 for the remaining 5% (poor match). We have find that for this network *optSampler* gives almost systematically a better approximation than *gpSampler*. We show in Fig 4 the KLD values of the marginal distributions of non null fluxes for *gpSampler* and *optGpSampler* compared with our hit–and–run implementation, ordered for increasing values, and some histograms representative of the aforementioned levels of approximation. For the largest network analyzed of Cervix squamous epithelial cells the level of approximation is worse, we refer to the supplementary materials. Finally, in order to test the consistency of ACHR methods on fully controlled instances we focused on sampling points from simple hypercubes of increasing dimensions. While on low dimensions we find a good agreement with an uniform distributions we report that ACHR based methods show some inconsistencies in higher dimensional instances. In Fig 5 (A) we show the distribution retrieved by the hit-and-run and by ACHR methods for the first coordinate of an hypercube of *D* = 500 after 2⋅10^7^ steps. In Fig 5 (B) we show the average KLD of the histograms of coordinates in the hypercube after 2⋅10^7^ montecarlo steps for gpsampler, optgpsampler and the hit-and-run as a function of the dimension respectively. We can see a crossover in both ACHR-based samplers for high dimension that is absent for the hit-and-run. In this case, at odds with the case of metabolic networks, it seems that gpsampler shows better performances than the optgpsampler, if they are referred to the same total number of montecarlo steps. However gpsampler is much slower than hit-and-run and optgpsampler, these two showing similar machine time per step e.g. the histograms in Fig 5 (A) have been obtained with gpsampler after one day, whereas the histograms for optgpsampler and the hit-and-run are obtained after 10 minutes on an intel dual core working at 3.06GHz (single thread). We thus report as well for sake of comparison the results of gpsampler for the same machine time.

**Fig 6:**
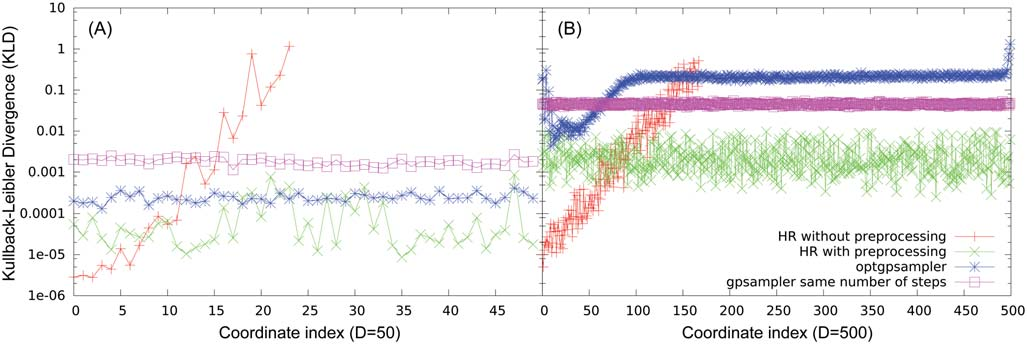
Consistency test of *optGpSampler* and *gpSampler* on heterogeneous hyper-rectangles. (A): KL divergence of the axis for HR, with and without preprocessing, *optGpSampler* and *gpSampler* (same number of steps, 2⋅10^7^) in *D* = 50. (B): KL divergence of the axis for the same methods in *D* = 500.

We have extended these analysis on heterogeneous hyper-rectangles of dimensions *D* = 50 and *D* = 500 whose axis length span from 10^−2^ to 10^5^. In Fig 6 we show the KL divergence with respect to the uniform distribution for all the axis obtained with the four methods, i.e. HR without preprocessing, HR with preprocessing, optgpsampler and gpsampler (same number of steps, 2⋅10^7^). The hit and run without ellipsoidal preprocessing is not able to sample efficiently along the larger axis, even for *D* = 50, The ellipsoidal preprocessing reduces the problem of hyper-rectangles to the problem of hypercubes that we have already described in detail, pointing out that in this case the HR is very efficient and controlled. The performances of ACHR-based methods, gpsampler and optgpsampler are slightly worst for hyper-rectangles with respect to the homogeneous case (hypercubes), but very similar. Once again, they correctly perform the sample in feasible machine times for *D* = 50, but they have problems for *D* = 500. This shows that ACHR-based methods somehow resolve the heterogeneity problem but they have problems in high dimensions.

## Materials and Methods

Given a *D*-dimensional convex set *P*, from which one wants to sample from, and a point inside *x*_*t*_ ∈ *P*, the standard HR algorithm is defined as follows:

1. Choose a uniformly distributed direction ***θ***_*t*_, that is, a point extracted from the uniform distribution on the *D*-dimensional unit sphere. This can be done with the Marsaglia method, i.e. by generating *D* independent gaussian random variables $\theta_{t}^{i}$ with zero mean and unit variance, and then normalizing the vector to unit length;
2. Extract *λ* ^⋆^ uniformly from the interval [*λ*_min_,*λ*_max_], where *λ*_min_ (*λ*_max_) is the minimum (maximum) value of *λ* such that **x**_*t*_+*λ* ***θ***_*t*_ ∈ *P*;
3. Compute the point **x**_*t*+1_ = **x**_*t*_+*λ* ^⋆^ ***θ***_*t*_, increment *t* by one and start again.

The starting point can be found, for instance, by interpolating between two vertices obtained by linear programming. The second step requires to find the intersections among a line and *P*. Since *P* is convex, the intersection points are only two, namely **x**_*t*_+*λ*_min_ ***θ***_*t*_ and **x**_*t*_+*λ*_max_ ***θ***_*t*_. Clearly, in order to perform the HR dynamics we should always use a *full-dimensional* representation of the convex set (see the supporting materials for further details); if not, *λ* ^⋆^ = *λ*_min_ = *λ*_max_ for almost all ***θ***_*t*_, and dynamics is frozen.

The decorrelation properties of the standard HR dynamics can be greatly improved by a slight modification of step 1, that is, extracting ***θ***_*t*_ from the surface of the matching ellipsoid instead of the unit sphere. This can be easily done by multiplying a random point on the unit sphere by the symmetric matrix which defines the ellipsoid, and normalizing the resulting vector to unit length. Below we describe three different methods (illustrated in Fig 1) in order to find or approximate the matching ellipsoid. We refer to the supporting materials for further details on the dynamics and the construction of the ellipsoid.

### Building the ellipsoid with PCA

If we had already solved the problem, that means we have a set of uniformly distributed independent points inside the polytope, we can use them to build a matching ellipsoid by Principal Component Analysis (PCA). The idea is that any sampling attempt of the polytope, even if not-equilibrating, it gathers some information on the form of the space. The connected covariance matrix from this sampling can be diagonalized and its eigenvalues and eigenvectors would give the axis of an ellipsoid that approximately matches the underlying space, the closer the nearer the sampling to equilibrium, in essence:

- Perform an HR markov chain up to time *T*, computing the covariance matrix of the sampled points.
- Diagonalize the connected covariance matrix and build an ellipsoid with axis along the principal components.
- Use the ellipsoid for the subsequent sampling.

The drawback of this procedure relies in the fact that ideally the sampling times *T* should be such that the covariance matrix attains stationarity and this convergence is slow if some preprocessing is lacking. We will see that for practical purposes PCA can be used to refine the results obtained by the more direct approaches that we describe in next sections.

### Building the ellipsoid with LP

If we would be able to calculate the diameter of the space, then find the diameter in the orthogonal space with respect to the previous diameter and so on, we would have a matching ellipsoid whose axis coincides with such diameters. Unfortunately, the calculation of the diameter of a convex closed space is a very hard task [28] (think to a randomly tilted hyper-rectangle) but we can recur to an approximation by performing what is called a Flux Variability Analysis [29] (FVA) in the field of metabolic network analysis. This consists in calculating the minimum and maximum values of each variable and this is a linear programming problem:

$$\begin{array}{ccc} & & \mathrm{Minimize}/\mathrm{Maximize}\;v_{i}\\ & & S\cdot v=0,\\ & & v_{r}\in \left[v_{r}^{\min},v_{r}^{\max}\right] \end{array}$$ (5)

that can be efficiently solved for instance with the simplex algorithm or by conjugate gradients methods. If we consider the vectors that go from the minimum to the maximum vertex for each variable, we take the vector of maximum length as the main axis of our ellipsoid and repeat FVA in the space orthogonal with respect to previous found axis, in synthesis:

- INPUT: The polytope P, a set of axis *U* = {**u**_1_, …,**u**_*k*_} for the ellipsoid E
- Perform FVA within the polytope P in the space orthogonal to the subspace generated by U. Take the vector **v** of maximum length connecting min and max vertices orthogonal to U.
- OUTPUT: **v** = **u**_*k*+1_ is a new axis for the ellipsoid E.

The good point of this procedure is that it is based on the resolution of well defined set of LP problems. Even if this procedure is polynomial and feasible, we have to solve a large number of linear programming problems (order 𝒪(*N* ^2^)). We have thus applied fast conjugate gradient techniques [30] as we describe in the supporting materials.

### The Lovazs ellipsoid method

We want to construct a couple of concentric ellipsoids *E*,*E* ^′^ matching the polytope *P*, i.e. such that *E* ^′^ ⊆ *P* ⊆ *E*, where *E* ^′^ is obtained from *E* shrinking by a factor 𝒪(1/*D* ^3/2^). This is called weak Loewner–John pair. We define a series of enclosing ellipsoids *E*_*k*_, starting with *E*_0_ as the sphere with center in the origin and radius *R* large enough in order to inscribe the body, according to the following lines:

- INPUT: An ellipsoid *E*_*k*_ with its center **x**_*k*_
- Check if **x**_*k*_ ∈ *P*, if yes go to 2, if no go to 1
- 1) Consider an hyperplane separating **x**_*k*_ and *P*, and the halfspace enclosing *P*, calculate the ellipsoid of minimal volume enclosing *H*∩*E*_*k*_ go to OUTPUT 1
- 2) Determine the endpoints of the axis of *E*_*k*_, shrink the ellipsoid and check if the shrinked ellipsoid $E_{k}^{\prime }$ is inside. if yes go to OUTPUT 2, if no go to 3
- 3) Consider an endpoint of an axis of the shrinked ellipsoid outside *P*, e.g. ${\text{x}}_{k}^{\prime }$, consider an hyperplane *H* separating ${\text{x}}_{k}^{\prime }$ and *P*, and the halfspace enclosing *P*, calculate the ellipsoid of minimal volume enclosing $H\cap E_{k}^{\prime }$ go to OUTPUT 1
- OUTPUT 1: A new ellipsoid *E*_*k*+1_ of lower volume with center **x**_*k*+1_, update *k*, repeat from INPUT.
- OUTPUT 2: A weak Loewner-John ellipsoid.

This algorithm is substantially an expanded version of the famous ellipsoid method used to demonstrate the feasibility of linear programming problem. Upon calculating the reduction in volume of the enclosing ellipsoid after one step, it can be demonstrated that this series converges in polynomial time to a weak Loewner–John pair. We refer to [21, 31] for further details.

## Conclusions

In this article we have proposed rounding methods in order to reduce the condition number for the application of the hit–and–run (HR) Markov Chain Monte Carlo to the problem of the uniform sampling of steady states in metabolic network models. They are based on matching the polytope under exam with an ellipsoid that can be used to bias the HR random walker, still sampling the flux space in a uniform way. Such ellipsoids were built by applying principle component analysis to previous sampling, by solving a set of linear programming problems—similarly to a technique called Flux Variability Analysis in the field of metabolic network analysis, and by the Lovazs ellipsoid method. In particular the last two can be calculated in polynomial times. We have applied them in order to sample the feasible steady state of three metabolic network reconstruction of growing size where we successfully removed the ill–conditioning and reduced dramatically the sampling times with respect to the normal HR dynamics. The Lovazs method or the LP method alone were sufficient to remove the ill–conditioning. With our implementation the first gives better results on the small network, the second on the genome scale networks, whereas the PCA can be used to refine the results of the other two, since in this case it is possible to obtain the ellipsoid from a diagonalization of a good estimator of the stationary connected covariance matrix. The overall procedure, preprocessing and subsequent sampling is feasible in genome scale networks, in agreement with theoretical results on the computational complexity regarding these tasks. The rounding preprocessing is still quite intensive on large genome scale models with our implementation and this leaves space for optimizing time performances that we leave for further investigations. Even if there could be faster methods in order to sample points inside convex polytopes, the HR Monte Carlo is guaranteed to converge to an uniform distribution. It could be used thus in order to test the correctness of fast message-passing [19] or ACHR-based algorithms in their convergence to an uniform distribution. We have thus compared the samples retrieved by the HR method with two ACHR based method provided with the COBRA toolbox *gpSampler* and *optGpSampler*. We checked that they generate similar distributions with rather different speed, *optGpSampler* being much faster. We have found that in the small network these ACHR-based methods are consistent with the uniform sampling provided by the hit an run according to the Kolmogorov-Smirnov test. On genome scale networks the flux distributions retrieved by these methods do not pass the KS test, but give an approximation that we quantified by calculating their Kullback Leibler divergence with respect to the distribution obtained with HR dynamics. In some cases we detected inconsistencies that get worse on higher dimensions. This behavior has been highlighted upon sampling high dimensional hypercubes. We want to mention finally that in regard to the problem of sampling the steady states of a metabolic network a rigorous implementation of thermodynamic constraints possibly renders the space non-convex [32]. The development of Montecarlo methods for the sampling of non convex spaces is a difficult open issue. The HR algorithm applied to the sampling of non–convex bodies is not guaranteed to converge in polynomial times, an aspect that needs further investigations.

## Supporting Information

S1 Fig Integrated autocorrelation times of the coordinate axis of a highly heterogeneous hyperrectangle in D = 20 sampled with hit-and-run dynamics with and without preprocessing. (EPS)

S2 Fig Autocorrelation function during ellipsoid-based hit-and-run markov chain Montecarlo of the 59th reaction flux calculated averaging over 2⋅10^5^ points. The signal-to-noise ratio decreases strongly when the function approaches zero, leading to a difficult numerical estimate of its integral (integrated autocorrelation time, inset).
(EPS)

S3 Fig Estimate of the integrated autocorrelation time of the function depicted in S2 Fig by binning the data. X axis: Bin length; Y axis: ratio of the variance of the binned data over the variance of unbinned data; error bars calculated from a gaussian approximation.
(EPS)

S4 Fig Histograms of the carbon dioxide excretion in SciND750 retrieved by the hit and run, optGpSampler and gpSampler, the latter upon waiting different times for convergence. (EPS)

S5 Fig Kullback-Leibler divergences of the marginal ux distributions obtained with optGpSampler with respect to the hit and run ordered for increasing values for the three metabolic networks examined. (EPS)
